# Psychometric properties of the Collective Efficacy Scale Short-Form in Chilean teachers

**DOI:** 10.3389/fpsyg.2022.935578

**Published:** 2022-10-13

**Authors:** Camilo Herrera, Javier Torres-Vallejos, Jonathan Martínez-Libano

**Affiliations:** ^1^Doctorado en Salud, Bienestar y Calidad de Vida, Facultat d’Educació i Psicologia, Universitat de Girona, Girona, Spain; ^2^Facultad de Educación y Ciencias Sociales, Universidad Andres Bello, Santiago, Chile

**Keywords:** teachers, collective efficacy, well-being, school context, factor analysis

## Abstract

**Background:**

The Collective Efficacy Scale Short-Form (CES-SF) is a short and reliable instrument that assesses collective efficacy in schools at a group level. Previous research has shown a two-factor structure considering the perception of the group competence about their teaching capabilities and task analysis that refers to the opportunities inherent to a specific task. However, there is no conclusive evidence that collective efficacy corresponds to a two-factor model or single-factor structure.

**Methods:**

A cross-sectional research was conducted on a 693 sample of teachers (*M*_*age*_ = 39.4; SD = 11.8) from schools in the 16 regions of Chile. They were assessed using the CES-SF, Personal Well-being Index, Social Well-Being Scale, and satisfaction with the school. Exploratory and confirmatory factor analyses were used to assess the construct validity of the CES-SF.

**Results:**

The CES-SF showed mixed results about its construct validity. Best fit has been found to retain two new factors (opportunities and challenges for collective efficacy) with eight items each, yielding a McDonald’s ω of 0.803. Convergent validity was also established.

**Conclusion:**

The psychometric results suggest that a two-factor structure for the CES-SF is a valid and reliable measure for this construct for Chilean teachers. However, collective efficacy might not strongly relate to subjective wellbeing but to school-context variables.

## Introduction

Collective efficacy refers to the shared belief within a group structure about their standard abilities related to the organization and execution of courses of action (Gurcay et al., 2009), thus extending the theory of efficacy from the individual level to the group organizational level (Bandura, 1986). The development of personal efficacy depends not only on individual assets but also on the social and institutional resources with which individuals come into contact (Reyes-Rodríguez et al., 2021). Belief in the capabilities of a group to organize and execute the courses of action required to achieve a goal is an essential organizational property because it facilitates goal attainment (Salloum, 2021). Thus, collective efficacy is the shared beliefs of group members about whether they can work together to achieve the goal of a specific task (Sun and Lin, 2022).

Collective efficacy has been correlated with various organizational outcomes, such as job satisfaction and burnout (Yurt, 2022), organizational belongingness, organizational commitment, and job wellbeing (Awuor et al., 2022; Gómez-Leal et al., 2022; Sánchez-Rosas et al., 2022). A recent systematic review found various personal, structural, group, process, and organizational factors (Butel and Braun, 2019). Individual factors include willingness or commitment to collaborate, understanding the benefits of teamwork, and the combination of particular skills, knowledge, and experience in teamwork. The most important structural factors are related to issues of time, continuity of personnel, physical proximity, and formalization/regulation of professional interaction. Group and process characteristics (i.e., the specific aspects of a particular team and its work together) had the most facilitators, including team size, supportive atmosphere, transformational leadership and flexibility, task emphasis, and interdependence (Vangrieken et al., 2015). Given the above, it is essential to have a measure of collective efficacy for teachers that allow addressing the construct in a specific way due to the different variables related to it to maximize the benefits of a school’s functioning.

### Collective efficacy in teachers

Collaboration among teachers has long been highly valued for its significant benefits, including increased motivation, job satisfaction, self-efficacy, and collective efficacy and its role in teacher professional development and school improvement (Swafford and Anderson, 2020; Bükki and Fehérvári, 2021).

From the education perspective, Gurcay et al. (2009) reported that students, teachers, and school administrators develop common beliefs that can be studied in terms of self-efficacy and act according to them. Collective teacher self-efficacy refers to the perceptions that a group of teachers in a school has about their ability to work together to generate a positive effect on their students (Goddard et al., 2000).

Studies have found that teacher collaboration can significantly foster professional learning and improve student achievement (Bolam et al., 2005; Goddard et al., 2015; Reeves et al., 2017). Thus, teachers’ collective efficacy predicts student success (Deltour et al., 2021), as outcomes are more effective when leading individuals in a community to embrace collectively valued goals rather than forcing them to do so (Peraza-Balderrama et al., 2021).

In educational settings, teachers’ perceptions of collective efficacy refer to a personal judgment of their colleagues’ abilities to perform instructional practices that support academic and psychosocial adjustment in school (Goddard et al., 2004) and in the collective ability of faculty members to positively affect student learning outcomes (Goddard et al., 2015).

### Collective efficacy measurement

After Gibson and Dembo’s (1984) teaching efficacy scale, designed to measure individual teaching efficacy, Goddard et al. (2000) created the Collective Efficacy Scale (CES). This scale considers a model with two dimensions of collective efficacy, namely, “group competence” and “task analysis,” reflecting perceptions of the group competence (GC) judged to the task. The CES is a 21-item scale that measures teachers’ collective efficacy based on the assumption that previous studies had considered measuring at the individual level (i.e., see Shamir et al., 2000; Ellemers et al., 2013), ignoring the effects of group membership (Goddard, 2002). This scale attempts to address this challenge by developing items that consider the judgment of the collective about the whole faculty (“Teachers in this school have what it takes to get the children to learn” instead of “I have what it takes to get my students to learn”). Later, Goddard (2002) tested a short form for the CES, considering 12 items more balanced than its 21-item version.

Other measures have been developed based on Goddard’s research. The Collective Teacher Efficacy Scale (EC-CTES; Donohoo et al., 2020) addresses advanced teacher influence, goal consensus, knowledge of others’ work, cohesive staff, leadership responsiveness, and Effective Systems of Intervention. Collective Teacher Self-Efficacy Scale (Sánchez-Rosas et al., 2021) is a self-report instrument that measures beliefs about the capabilities of the teaching team. Specifically, it was designed to assess, using 45 items, six dimensions of collective teacher self-efficacy: self-efficacy for decision-making (8 items), self-efficacy for teaching (8 items), self-efficacy for coexistence (7 items), self-efficacy for family involvement (7 items), self-efficacy for community involvement (7 items), and self-efficacy for positive school climate (8 items).

### The present study

There is no consensus about measuring collective efficacy in schools considering one or several related factors. This study aimed to test the factor structure of collective efficacy that applies to teachers in Chilean schools. The chosen measure is the 12-item Collective Efficacy Scale Short-Form (CES-SF) developed by Goddard (2002), which is a short and widely used in different cultures measure for collective efficacy (Goddard et al., 2004; Baleghizadeh and Goldouz, 2016). We hypothesized that (1) the CES-SF might retain its two-factor structure and (2) there will be a positive relationship between the validated measure of CES-SF and scales of subjective wellbeing, satisfaction with the school, and social wellbeing at school.

## Materials and methods

### Participants

This study used a probabilistic and stratified sample of schools in the different urban zones of the 16 regions of Chile in 2018. The sampling framework was the 2017 National School Enrollment Registry from the Chilean Ministry of Education. There were 693 teachers, and the mean age was 39.4 years (*SD* = 11.8). Most of the teachers were from public schools (44.8%) and subsidized schools (42.4%), followed by private ones (11.1%) and another administrative dependency (1.8%). According to the National School Vulnerability Index (IVE-SINAE, known as the school vulnerability percentage, which corresponds to the percentage of students in a situation of social vulnerability), 50% of the schools catered to students with low SES, 25.4% with medium SES and 24.6% with high SES.

### Measures and instruments

*Collective Efficacy Scale Short-Form* (Goddard, 2002). Based on its original 21-item version, this scale included 12 items rated on a 6-point scale (1 = “strongly disagree”; 6 = “strongly agree”). Different items reflected two dimensions, namely, GC and task analysis (TA), each with six items, and both positively and negatively worded items appeared.

### Criterion variables

*Personal Wellbeing Inventory for Adults* (PWI-A) was originally developed by The International Wellbeing Group (2013) and adapted for the Chilean context by Oyanedel et al. (2015). This scale measures subjective wellbeing considering seven dimensions of satisfaction (one for each item) and two additional items related to religion or spirituality (Wills, 2009; Żemojtel-Piotrowska et al., 2017) and overall life satisfaction (Campbell et al., 1976). This 9-item scale is rated on a 11-point scale (0 = “Completely dissatisfied”; 10 = “Completely satisfied”). For this sample, the internal consistency for the full scale was ω = 0.900.

*Satisfaction with school* is a 6-item scale created to evaluate different aspects of satisfaction with the school as an institution. It also asks about the relationships between various educational community members, whether they would recommend this school to others, and whether they like it. The internal consistency for the 6-item scale was ω = 0.893.

*Social wellbeing at school scale* (SWS) is a scale created by Keyes (1998) and adapted to school context and teachers by Bilbao et al. (in press) that assesses five dimensions of social wellbeing, contextualizing their evaluation of their school as a context-based experience of school as a society. The adapted version of 22 items had a 5-point rating scale (1 = “completely disagree”; 5 = “completely agree”). The internal consistency for the full scale was ω = 0.851.

### Procedure and ethical considerations

This study is part of a larger investigation carried out by the Research Center for Inclusive Education of the *Pontificia Universidad Católica de Valparaíso*, Chile, where different scales were applied to students, teachers, parents, and management teams in order to characterize educational trajectories. However, for this particular research, we worked only with data from teachers.

Participation in this study was supported by the signature of the researcher and participant of the letter of consent following the regulations of the Ethics Committee of Pontificia Universidad Católica de Valparaíso, Chile, following de Declaration of Helsinki. All participants signed informed consent forms. All questionnaires were administered in the schools where participants worked. This research was approved by the Ethics Committee of Pontificia Universidad Católica de Valparaíso, Chile under the code BIOEPUCV-H 427-2021.

### Statistical analysis

First, negatively worded items were reversed before the calculation of later analyses. A descriptive and correlation analysis was used for the items of the CTES-SF. Later, two types of factor analyses were performed: first, to explore how the items related to each other (exploratory factor analysis, EFA), and second, to confirm its theoretical structure and other obtained for this sample by EFA (confirmatory factor analysis, CFA). The reliability of the CES-SF was evaluated using McDonald’s omega (ω), considering acceptably reliable coefficient values greater than 0.90 (McDonald, 1999).

The EFA used the robust factor analysis with Diagonally Weighted Least Squares (DWLS), polychoric correlation matrices, and promin rotation (Lorenzo-Seva and Ferrando, 2019). The parallel analysis test was used to determine the most appropriate number of dimensions (Timmerman and Lorenzo-Seva, 2011) and the closeness to unidimensionality assessment (Ferrando and Lorenzo-Seva, 2018) with the convergence of three indices, namely, UniCo, ECV, and MIREAL, to determine its unidimensional structure.

Confirmatory factor analysis was performed using robust weighted least square mean and variance adjusted (WLSMV), considering the ordinal nature of the response rating scale. The models evaluated in the confirmatory analysis were those theoretically proposed by Goddard (2002) and later those produced by EFA. The evaluation of the different models was performed considering other goodness-of-fit indices (GFIs): the comparative fit index (CFI with appropriate values ≥0.90; the standardized root mean square residual (SRMR); and the root means square error of approximation (RMSEA), with a confidence interval of 90%, both with adequate values <0.08 (Hu and Bentler, 1999).

Finally, the convergent validity of the most appropriate model was evaluated using correlation analyses between CES-SF and PWI-A, satisfaction with school, and SWS scales. Correlations were expected to be positive moderately or strongly related between the different measures.

All the analyses were performed using Factor version 12.01.02 (Ferrando and Lorenzo-Seva, 2017) and MPlus version 8.7 Base Program and Combination Add-On (Muthén and Muthén, 1998–2007).

## Results

### Descriptive analysis

To characterize the items of the CES-SF for teachers and the relationships between them, descriptive statistics – including mean and standardized deviations – and the correlation matrix are summarized in Table 1. Most of the items show positive and significant correlations among themselves.

**TABLE 1:** Descriptive statistics and correlation matrix for Collective Efficacy Scale Short-Form (CES-SF) (*n* = 693).

	1	2	3	4	5	6	7	8	9	10	11	12
Item 1	–											
Item 2	0.52***	–										
Item 3 (r)	0.26***	0.46***	–									
Item 4 (r)	0.29***	0.35***	0.49***	–								
Item 5	0.53***	0.43***	0.25***	0.32***	–							
Item 6	0.09*	0.37***	0.16***	0.07	0.22***	–						
Item 7	0.06	0.15***	0.03	−0.07	0.05	0.51***	–					
Item 8 (r)	0.15***	0.09*	0.20***	0.23***	0.16***	0.18***	0.17***	–				
Item 9 (r)	0.29***	0.46***	0.48***	0.51***	0.30***	0.20***	−0.04	0.20***	–			
Item 10	0.36***	0.48***	0.23***	0.24***	0.44***	0.33***	0.23***	0.09*	0.24***	–		
Item 11 (r)	0.11**	0.20***	0.31***	0.28***	0.17***	0.07	−0.11**	0.16***	0.36***	0.09*	–	
Item 12 (r)	0.14***	0.15***	0.21***	0.16***	0.19***	0.17***	0.10*	0.24***	0.21***	0.08*	0.37***	–
*M*	4.93	4.88	4.61	4.88	5.20	3.48	2.85	3.79	4.74	4.65	5.06	4.67
SD	0.97	1.05	1.31	1.29	1.02	1.36	1.47	1.32	1.30	1.16	1.17	1.50

***p < 0.001, **p < 0.01, *p < 0.05. (r), reversed item.

### Exploratory factor analysis and reliability estimates

Exploratory factor analysis was used for item reduction as well as to understand the underlying factor structure (Table 2). The analysis was shown to be excellent for the overall sample, with a KMO of 0.814 and a statistically significant Bartlett’s test of sphericity (*p* < 0.001). The highest reported communality was reported for item 2 (0.954) and the lowest for item 7 (0.276). Results do not suggest a unidimensional structure (UniCo = 0.864; ECV = 0.754; MIREAL = 0.246). Then, the two-factor solution showed that items 6 and 7 configure a second factor. All items were strongly loaded on their respective factors, except for items 8, 11, and 12. The three-factor solution showed that items 6 and 7 loaded into a different factor, and item 8 loaded poorly to its factor. These three solutions suggest the removal of items 6, 7, and 8 for the final factor structure.

**TABLE 2:** Factor loadings of exploratory factor analysis with the one, two, and three factor solutions (*n* = 693).

		One-factor solution	Two-factor solution		Three-factor solution		
				
	Theoretical factors	λ F1	λ F1	λ F2	λ F1	λ F2	λ F3
Item 1	Group competence	0.644	0.637	–	0.899	–	–
Item 2	Group competence	0.796	0.769	–	0.744	–	–
Item 3 (r)	Group competence	0.691	0.714	–	–	–	0.583
Item 4 (r)	Group competence	0.692	0.732	–	–	–	0.587
Item 5	Group competence	0.725	0.714	–	0.808	–	–
Item 6	Task analysis	0.407	0.325	0.609	–	0.685	–
Item 7	Task analysis	0.201	–	0.726	–	0.751	–
Item 8 (r)	Task analysis	0.324	0.318	–	–	–	0.383
Item 9 (r)	Group competence	0.729	0.758	–	–	–	0.613
Item 10	Task analysis	0.636	0.596	0.316	0.704	–	–
Item 11 (r)	Task analysis	0.460	0.492	–	–	–	0.768
Item 12 (r)	Task analysis	0.399	0.401	–	–	–	0.595
McDonald’s ω		0.767	0.783	–	0.769	–	0.710

Loadings lower than absolute 0.300 were omitted.

### Confirmatory factor analyses

Confirmatory factor analysis was used to confirm the different solutions obtained from the EFA analysis (Table 3). The assumption of a global collective efficacy (one-factor models, Models 1 and 2 in Table 3) was compared with its multidimensional structure (two-factor models, Models 3–8 in Table 3), considering the prior removal of items 6, 7, and 8. One-factor models (Models 1 and 2) and two-factor models (Models 3 and 4), both theoretically proposed, showed poor fit to the data, even adding two error covariances for each model. Then, the two-factor proposed model, based on the three-factor solution in Table 2, showed a better fit removing item 12 that showed the lower factor loadings and adding one covariance between items 1 and 5 errors.

**TABLE 3:** Goodness-of-fit indices of alternative confirmatory factor analysis (CFA) models.

Model	χ^2^ (df)	CFI	RMSEA [LCI, UCI]	SRMR	Model description
1	698.889*** (27)	0.894	0.189 [0.177, 0.202]	0.063	One-factor model, w/o items 6, 7, 8
2	442.661*** (25)	0.934	0.155 [0.143, 0.168]	0.047	One-factor model, w/o items 6, 7, 8 + 2 error covariances (i11↔i12; i1↔i5)
3	696.865*** (26)	0.894	0.193 [0.181, 0.205]	0.063	Two correlated factors theoretical model, w/o i6, i7, i8
4	439.693*** (24)	0.934	0.158 [0.145, 0.171]	0.046	Two correlated factors theoretical model, w/o i6, i7, i8 + 2 error covariances (i11↔i12; i1↔i5)
5	299.474*** (26)	0.957	0.123 [0.111, 0.136]	0.041	Two-factor proposed model (Table 2) w/o i6, i7, i8
6	149.617*** (25)	0.980	0.085 [0.072, 0.098]	0.025	Two-factor proposed model (Table 2) w/o i6, i7, i8 + 1 error covariance (i11↔i12)
7	159.482*** (19)	0.977	0.103 [0.089, 0.118]	0.026	Two-factor proposed model w/o i6, i7, i8, i12
8	109.117*** (18)	0.985	0.085 [0.070, 0.101]	0.022	Two-factor proposed model w/o i6, i7, i8, i12 + 1 error covariance (i1↔i5)

***p < 0.001. GC, group competence; TA, task analysis.

**FIGURE 1:**
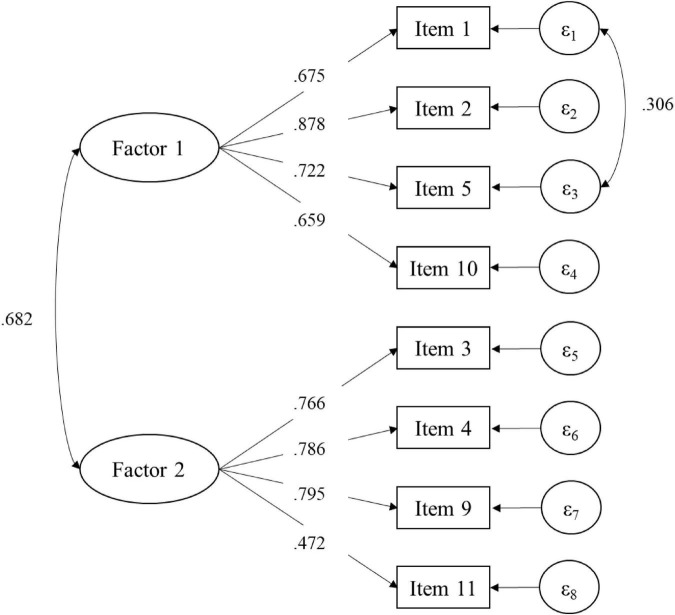
Factorial loadings of the Model 8 in Table 3 (*n* = 693).

Model 8 (Figure 1), with eight items (ω = 0.803), presented the best fit to the data where Factor 1 (ω = 0.769) represents opportunities and conditions for collective efficacy and Factor 2 is related to challenges for collective efficacy (ω = 0.745).

These two new definitions for the found factors arise from the association of items, where factor one involves elements that teacher controls. In contrast, the second factor focuses on features that teachers need to overcome to be effective.

### Construct validity

The external construct validity was tested by implementing correlations with other tests that were theoretically correlated with collective efficacy. A correlation analysis was performed on each scale to test the CES-SF’s construct validity for the teachers’ sample (Table 4). The results showed that the CES-SF is related positively to other criterion variables: it yielded a statistically significant but weak correlation with the PWI-A (subjective wellbeing) but higher with satisfaction with the school (*r* = 0.518, *p* < 0.001) and social wellbeing at school (*r* = 0.564, *p* < 0.001). In both cases, the opportunities dimension was strongly correlated with each criterion variable and the challenges factor.

**TABLE 4:** Correlations between CES-SF and comparison variables.

	CES-SF		
	
	F1 Opportunities	F2 Challenges	Total score
**CES-SF**			
Factor 1: opportunities	–		
Factor 2: challenges	0.237***	–	
Total score	0.815***	0.756***	–
PWI-A	0.316***	0.299***	0.391***
Satisfaction with the school	0.500***	0.275***	0.501***
Social wellbeing at school	0.484***	0.361***	0.541***

***p < 0.001.

## Discussion

This study aimed to test the factor structure of the CES by Goddard (2002) among Chilean teachers. The analysis revealed that this instrument might have a two-factor structure in the Chilean teachers’ sample, considering some modifications concerning its original composition, better than a unidimensional factor structure. First, when the EFA was performed, one, two, and three-factor solutions could not be configured like the original factors of the scale. Items 6, 7, 8, and 12 loaded poorly to their respective factors. We hypothesized that the poor performance of these items is because they refer to factors external to the community (Lauder et al., 2003), being beyond the faculty’s responsibility, and perhaps not part of collective efficacy itself.

Collective efficacy beliefs are essential factors in predicting psychological (Roos et al., 2013) and subjective wellbeing (Salanova et al., 2003). Results also showed that it is correlated to satisfaction with the school and social wellbeing at school, which are important school-context-related variables (López et al., 2021).

This tool can assess collective efficacy among teachers in the school context as a reliable and valid instrument. Although few studies validate the scale (Sánchez-Rosas et al., 2021) and others create a new scale based on it (Donohoo et al., 2020; Kapat et al., 2022), there is no consensus on its dimensionality. Also is shorter than the previous version, which is especially important in school contexts where there is less time to participate in studies.

As projections of this study, it would be advisable to have a larger sample to verify its invariance in different groups of interest, such as the type of school, educational level, and the school’s capabilities to guide its change processes and promote student learning. Bifactor analysis would be essential to determine the specific contribution of each item to a specific and a general factor, with variables to explain these differences. One of the limitations of this study has been to have a sample that does not allow us to distinguish between groups that are comparable and that have characteristics that can influence the obtaining of different factorial solutions.

## Data availability statement

The raw data supporting the conclusions of this article will be made available by the authors, without undue reservation.

## Ethics statement

This research was approved by the Ethics Committee of Pontificia Universidad Católica de Valparaíso, Chile under the code BIOEPUCV-H 427-2021. The patients/participants provided their written informed consent to participate in this study.

## Author contributions

CH conceptualized this study and chose the theoretical framework and measures. CH and JT-V designed the general study and the methods to be implemented. JT-V wrote several sections of the initial draft, carried out the analysis, and interpreted results. JM-L contributed to the literature review. All authors listed have made a substantial, direct, and intellectual contributions to this study, reviewed and drafted sections of the initial draft, interpreted the results, wrote, read, and revised the final manuscript, and approved it for publication.
